# A Million Years of Mammoth Mitogenome Evolution

**DOI:** 10.1093/molbev/msaf065

**Published:** 2025-04-09

**Authors:** J Camilo Chacón-Duque, Jessica A Thomas Thorpe, Wenxi Li, Marianne Dehasque, Patricia Pečnerová, Axel Barlow, David Díez-del-Molino, Kirstin Henneberger, Chenyu Jin, Kelsey N Moreland, Johanna L A Paijmans, Tom van der Valk, Michael V Westbury, Flore Wijnands, Ian Barnes, Mietje Germonpré, Elizabeth Hall, Susan Hewitson, Dick Mol, Pavel Nikolskiy, Mikhail Sablin, Sergey Vartanyan, Grant D Zazula, Anders Götherström, Adrian M Lister, Michael Hofreiter, Peter D Heintzman, Love Dalén

**Affiliations:** Centre for Palaeogenetics, Stockholm, Sweden; Department of Zoology, Stockholm University, Stockholm, Sweden; Department of Archaeology and Classical Studies, Stockholm University, Stockholm, Sweden; Wellcome Sanger Genome Institute, Cambridge, UK; Centre for Palaeogenetics, Stockholm, Sweden; Globe Institute, University of Copenhagen, Copenhagen, Denmark; Centre for Palaeogenetics, Stockholm, Sweden; Department of Zoology, Stockholm University, Stockholm, Sweden; Department of Organismal Biology, Uppsala University, Uppsala, Sweden; Department of Biology, University of Copenhagen, Copenhagen, Denmark; School of Environmental and Natural Sciences, Bangor University, Bangor, UK; Centre for Palaeogenetics, Stockholm, Sweden; Department of Zoology, Stockholm University, Stockholm, Sweden; Adaptive Evolutionary Genomics, Institute of Biochemistry and Biology, Faculty of Science, University of Potsdam, Potsdam, Germany; Centre for Palaeogenetics, Stockholm, Sweden; Department of Zoology, Stockholm University, Stockholm, Sweden; Department of Bioinformatics and Genetics, Swedish Museum of Natural History, Stockholm, Sweden; Centre for Palaeogenetics, Stockholm, Sweden; Department of Zoology, Stockholm University, Stockholm, Sweden; School of Environmental and Natural Sciences, Bangor University, Bangor, UK; Evolutionary Ecology Group, Department of Zoology, University of Cambridge, Cambridge, UK; Centre for Palaeogenetics, Stockholm, Sweden; Department of Zoology, Stockholm University, Stockholm, Sweden; Department of Bioinformatics and Genetics, Swedish Museum of Natural History, Stockholm, Sweden; Globe Institute, University of Copenhagen, Copenhagen, Denmark; Centre for Palaeogenetics, Stockholm, Sweden; Department of Geological Sciences, Stockholm University, Stockholm, Sweden; Natural History Museum, London, UK; Royal Belgian Institute of Natural Sciences, Brussels, Belgium; Government of Yukon Territory, Palaeontology Program, Whitehorse, Yukon, Canada; Government of Yukon Territory, Palaeontology Program, Whitehorse, Yukon, Canada; Natural History Museum Rotterdam, Rotterdam, The Netherlands; Russian Academy of Sciences, Geological Institute, Moscow, Russia; Russian Academy of Sciences, Zoological Institute, Saint Petersburg, Russia; Far East Branch, Russian Academy of Sciences, North-East Interdisciplinary Scientific Research Institute N.A. Shilo, Magadan, Russia; Government of Yukon Territory, Palaeontology Program, Whitehorse, Yukon, Canada; Centre for Palaeogenetics, Stockholm, Sweden; Department of Archaeology and Classical Studies, Stockholm University, Stockholm, Sweden; Natural History Museum, London, UK; Adaptive Evolutionary Genomics, Institute of Biochemistry and Biology, Faculty of Science, University of Potsdam, Potsdam, Germany; Centre for Palaeogenetics, Stockholm, Sweden; Department of Geological Sciences, Stockholm University, Stockholm, Sweden; Centre for Palaeogenetics, Stockholm, Sweden; Department of Zoology, Stockholm University, Stockholm, Sweden; Department of Bioinformatics and Genetics, Swedish Museum of Natural History, Stockholm, Sweden

**Keywords:** deep-time DNA, palaeogenomics, mammoths, mitogenomes, phylogenetics, molecular clock dating

## Abstract

The genomic study of specimens dating to the Early and Middle Pleistocene (EP and MP), a period spanning from 2.6 million years ago (Ma) to 126 thousand years ago (ka), has the potential to elucidate the evolutionary processes that shaped present-day biodiversity. Obtaining genomic data from this period is challenging, but mitochondrial DNA, given its higher abundance compared to nuclear DNA, could play an important role to understand evolutionary processes at this time scale. In this study, we report 34 new mitogenomes, including two EP and nine MP mammoth (*Mammuthus* spp.) specimens from Siberia and North America and analyze them jointly with >200 publicly available mitogenomes to reconstruct a transect of mammoth mitogenome diversity throughout the last million years. We find that our EP mitogenomes fall outside the diversity of all Late Pleistocene (LP) mammoths, while those derived from MP mammoths are basal to LP mammoth Clades 2 and 3, supporting an ancient Siberian origin of these lineages. In contrast, the geographical origin of Clade 1 remains unresolved. With these new deep-time mitogenomes, we observe diversification events across all clades that appear consistent with previously hypothesized MP and LP demographic changes. Furthermore, we improve upon an existing methodology for molecular clock dating of specimens >50 ka, demonstrating that specimens need to be individually dated to avoid biases in their age estimates. Both the molecular and analytical improvements presented here highlight the importance of deep-time genomic data to discover long-lost genetic diversity, enabling better assessments of evolutionary histories.

## Introduction

Ancient DNA (aDNA) recovered from specimens dated to the Early Pleistocene (EP; 2.6 Ma to 780 ka) and Middle Pleistocene (MP; 780 to 126 ka) stages—from here on referred to as deep-time DNA—has the potential to allow the direct study of the deep-time evolutionary events that are key to understanding species formation (Dalén et al. 2023). Unfortunately, access to deep-time DNA is limited, and so far only a handful of studies have been able to obtain either genome-wide data or complete mitochondrial genomes (mitogenomes) from deep-time specimens (Dabney et al. 2013; Orlando et al. 2013; Meyer et al. 2016; Froese et al. 2017; Karpinski et al. 2020; Barlow et al. 2021; van der Valk et al. 2021; Lord et al. 2022).

The oldest genome-wide DNA data ever recovered comes from two mammoths (*Mammuthus* spp.) that were dated to 1.6–1.1 Ma (van der Valk et al. 2021). These two individuals, Krestovka and Adycha, are morphologically indistinguishable from European steppe mammoths (*M. trogontherii*), but their genomes revealed two divergent lineages that coexisted in northern Siberia during the EP. One of these lineages, Adycha, is ancestral to woolly mammoths (*M. primigenius*), while a hybridization event during the MP between the Krestovka lineage and woolly mammoths gave rise to the Late Pleistocene (LP) Columbian mammoth (*M. columbi*) lineage in North America (van der Valk et al. 2021). These results showcase the potential of deep-time genomes to yield insights that cannot be directly assessed from morphological studies, such as the relationships among morphospecies and deep-time hybridization events.

Mitogenomes are generally more accessible in ancient or degraded samples compared to nuclear genomes due to their high copy number. They therefore offer a unique opportunity to increase spatiotemporal sampling of deep-time specimens, which may provide further insight into the early stages of species evolution. To date, only three complete deep-time mammoth mitogenomes have been recovered: two EP specimens (Krestovka, Adycha) and an MP woolly mammoth from northeast Siberia (Chukochya; dated to ∼700 ka). Combined with ∼200 publicly available LP mammoth mitogenomes, phylogenetic analysis indicates that the two EP mammoths fall outside MP and LP mammoth mitogenome diversity, in line with results from nuclear genomes (van der Valk et al. 2021).

Mitogenome diversity of LP mammoths is characterized by the existence of three major and deeply divergent clades (Barnes et al. 2007; Debruyne et al. 2008; Palkopoulou et al. 2013). The split between Clade 1 and the lineage leading to Clades 2 and 3 represents the earliest divergence within LP mammoth diversity. While Clade 2 is only known from Siberia, Clade 3 was once widespread across Europe, Siberia, and North America (Palkopoulou et al. 2013; Chang et al. 2017). Furthermore, the only MP mammoth mitogenome reported to date, Chukochya, has a basal position in Clade 3, suggesting a Siberian origin of this clade (van der Valk et al. 2021). Despite its early divergence, Clade 1 is so far only represented by LP individuals. Given its wide distribution in North America, it has been suggested that Clade 1 may have originated there, then expanded westwards across the Bering land bridge, leading to the replacement of both Clade 2 (by ca. 40 ka) and Clade 3 (by ca. 24 ka) in Eurasia (Debruyne et al. 2008; Palkopoulou et al. 2013).

Clade 1 was consequently the last remaining mammoth mitochondrial clade, and was widespread across the northern hemisphere from the height of the last glaciation (24 ka) until the final extinction of mammoths on Wrangel Island ca. 4 ka (Palkopoulou et al. 2013; Dehasque et al. 2021). Moreover, Columbian mammoths with available mitogenomes are a monophyletic sister group to Clade 1 woolly mammoths (Enk et al. 2016). As the Columbian mammoth is known to be a hybrid (van der Valk et al. 2021), this suggests that the woolly mammoth ancestors of Columbian mammoths likely belonged to Clade 1.

Additional deep-time mitogenomes could shed light on different aspects of mammoth evolutionary history and past population dynamics, such as the spatiotemporal origin and the potentially wider distribution of each clade during the MP. However, a major limitation of deep-time DNA for phylogenetic studies is the general lack of reliable and precise sample age information. This is due to the limit of radiocarbon dating being ∼50 ka (Reimer et al. 2020) such that, for older specimens, only broad date ranges can be provided by stratigraphic context data, if such context is available at all. To address this issue, many genetic studies involving specimens beyond the radiocarbon limit instead rely on molecular dating, implementing a Bayesian approach to infer age based on the molecular clock (Shapiro et al. 2011). However, varied implementation of this approach translates into a lack of a reproducible methodology to obtain reliable molecular age estimates. Overcoming this limitation is essential to accurately infer evolutionary histories in deep time and to be able to cross-compare studies.

Here we report 34 new mammoth mitogenomes, including 11 of deep-time age, and reconstruct a transect of mitogenome evolution across the last million years. Also, building upon previous methodologies, we further improve the current framework for molecular clock dating beyond the radiocarbon limit, demonstrating that specimens need to be individually dated to avoid a bias in the age estimates. Our deep-time study provides new insights into mammoth mitogenome diversification and evolution and can serve as a template for future research on other extinct and extant species.

## Results and Discussion

### A Catalogue of Deep-time Mammoth Mitogenomes

**Fig. 1.**
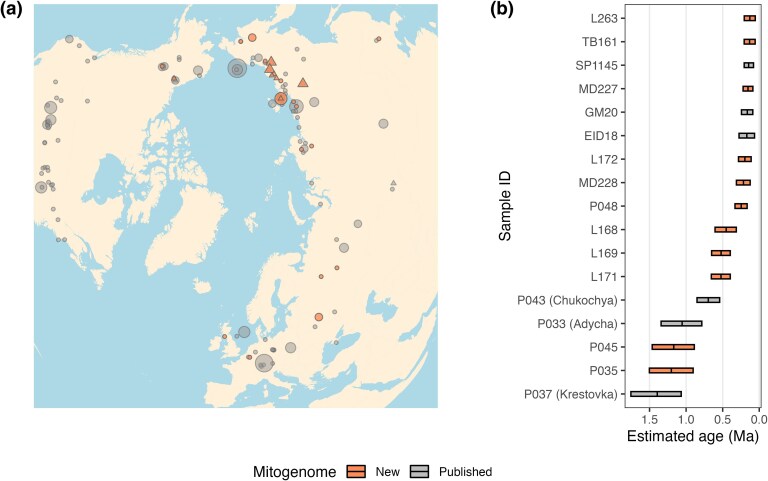
a**)** Geographic origin of mammoth specimens analyzed in this study (*N* = 225), including previously published and new mitogenomes. Triangles indicate deep-time (>126 ka) mitogenomes, as inferred by our molecular clock dating analyses. Shape sizes indicate the number of individuals sampled in the same location. The map has an orthographic projection plotted with the R packages: mapproj and ggmap. Detailed information on specimen localities can be found in supplementary tables S1 and S2, Supplementary Material online. b**)** Age estimates of deep-time mitogenomes (*N* = 17). Each bar displays the 95% Highest Posterior Density (HPD) interval, and the black line shows the mean estimate. The mitogenomes from the three specimens published by van der Valk et al. (2021) are shown as P037 (Krestovka), P033 (Adycha), and P043 (Chukochya). Tip-dating estimates are found in supplementary table S3, Supplementary Material online.

We generated 34 mitogenomes from mammoth specimens that have been either stratigraphically or radiocarbon dated to various stages of the Pleistocene from locations across the Northern Hemisphere (Fig. 1a, supplementary table S1, Supplementary Material online). Twenty-five of these were directly radiocarbon dated in previous studies (Hagelberg et al. 1994; Barnes et al. 2007; Palkopoulou et al. 2013; Martinez De La Torre et al. 2019), with 12 yielding dates beyond the limit of radiocarbon dating (>50 ka; non-finite). The remaining nine undated specimens were considered beyond the radiocarbon limit based on stratigraphic context (supplementary table S1, Supplementary Material online), some of which could not be clearly distinguished as showing either steppe mammoth or woolly mammoth morphology.

### Consistent Age Estimation of Mammoth Mitogenomes

We estimated the age of the undated specimens from their mitogenomes using a Bayesian phylogenetic approach for molecular clock tip dating (Shapiro et al. 2011), following the methodology implemented by van der Valk et al. (2021). This methodology included the use of a joint molecular clock analysis for estimating all unknown tip dates simultaneously (from here on referred to as “multi-sample dating”). However, another recent study on American mastodon (*Mammut americanum*) mitogenomes described a bias toward older and lower-precision estimates when using multi-sample tip dating compared to dating each sample independently—from here on referred to as “single-sample dating” (Karpinski et al. 2020). Based on these findings, and that the estimated tip dates for the three deep-time mammoth mitogenomes reported by van der Valk et al. (2021) are older than the stratigraphic record suggests, we sought to evaluate and implement the single-sample dating approach on these mitogenomes.

First, we implemented single-sample dating using the same parameters and multiple sequence alignment presented in van der Valk et al. (2021). The three deep-time specimens showed a closer agreement between the new single-sample estimated molecular tip dates and geological ages derived from stratigraphic context, as compared to estimates from the original multi-sample dating (supplementary fig. S1, Supplementary Material online). We next tested the effect of sequentially increasing the number of the oldest undated specimens in a given analysis from one to five. We found a directional bias toward older estimates with the inclusion of additional undated specimens (supplementary fig. S2, Supplementary Material online). Based on both findings, we chose to implement single-sample dating for age estimation of all undated mitogenomes. To avoid making assumptions about sample age in the absence of stratigraphic context, we used uniform age priors (see also supplementary Text S1, Supplementary Material online).

We then supplemented the dataset of van der Valk et al. (2021) with our 34 new and 21 previously published mitogenomes (Methods; supplementary table S2, Supplementary Material online), leading to a final dataset of 225 mammoth mitogenomes. We selected all mitogenomes with a finite radiocarbon date as tip calibration reference samples for single-sample dating, which included five of the new mitogenomes. We excluded eight of our new mitogenomes with known radiocarbon ages, which we used to examine the accuracy of our single-sample dating approach. We found that median calibrated radiocarbon estimates generally fall within the 95% High Posterior Density (HPD) ranges of the tip-dating estimates and, in most cases, are consistent with the mean tip dates. However, considering that the 95% HPD ranges are wide, we advise against using molecular clock dating as an alternative to radiocarbon dating for samples that can be finitely radiocarbon dated (supplementary Text S2, Supplementary Material online). Finally, all mitogenomes without a finite radiocarbon date were single-sample dated with tip dates converging to a uni-modal posterior distribution (supplementary table S3, Supplementary Material online).

Using the single-sample dating approach, we recovered a total of 17 deep-time mammoth mitogenomes, six of which have been reported as such previously (Fig. 1b, supplementary table S3, Supplementary Material online). The 11 new deep-time mitogenomes comprise two EP mammoths (P035, P045; > 1 Ma) and nine MP mammoths. Nine of these specimens have inferred ages based on their stratigraphic contexts that closely match the inferred tip dates, further validating the accuracy of our tip dating approach (supplementary Text S1, Supplementary Material online). Interestingly, three MP mammoths (L168, L169, and L171) date to ∼500 ka (95% HPD range between ∼300 and ∼650 ka). These samples have previously been estimated to be less than half this age (∼200 ka) based on a short fragment of the mitochondrial control region (Palkopoulou et al. 2013), confirming that, in some cases, using this region for molecular clock dating can lead to erroneous estimates of divergence times and sample ages, as proposed by early studies in the field (Ingman et al. 2000). Nonetheless, these limitations can be overcome by carefully choosing and validating (e.g. with simulated data) the correct parameters and priors for Bayesian inference (Shapiro et al. 2011). Additionally, we report 24 LP mammoths that are single-sample dated to >50 ka, including six that are new (supplementary fig. S3, Supplementary Material online, supplementary table S3, Supplementary Material online).

### Expanded Mammoth Phylogeography and Evolution

**Fig. 2.**
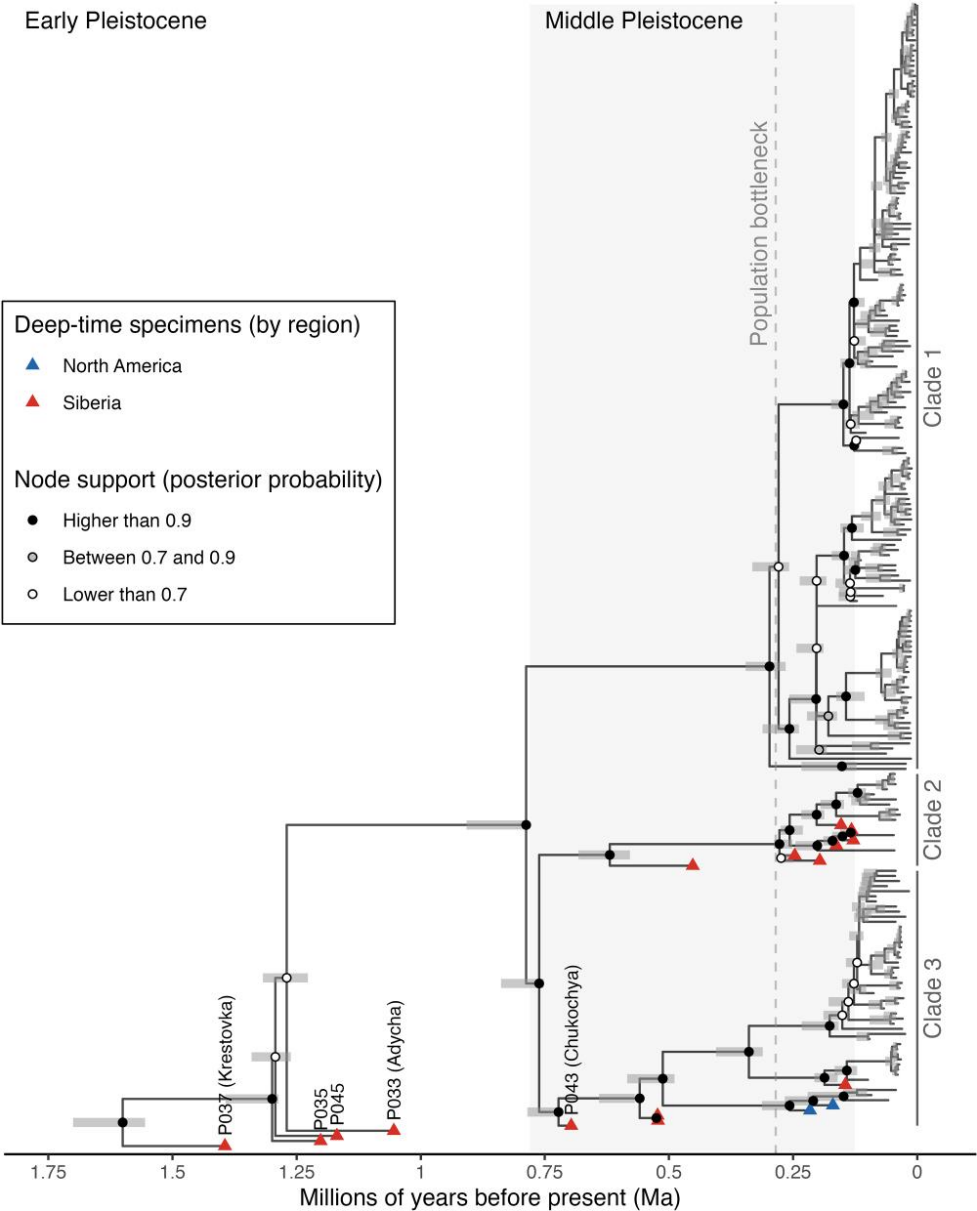
Joint phylogeny using single-sample dating estimates as input for tip ages. The original tree was built with *Elephas maximus*, *Loxodonta africana*, and *Loxodonta cyclotis* as outgroups (supplementary table S2, Supplementary Material online), but only mammoths were plotted for visualization purposes. The gray-shaded area represents the Middle Pleistocene epoch and the dashed line the mean estimate for the demographic bottleneck inferred with genome-wide data (Palkopoulou et al. 2015). The dark gray shades in the nodes display the 95% HPD node heights estimated by the Bayesian analyses. For visualization purposes, only the three deep-time mammoths from van der Valk et al. (2021) and our new Early Pleistocene mitogenomes are labeled in the tree, while the rest of the deep-time specimens are highlighted with a triangle (by geographical region). Labels for all samples are found in supplementary figs. S4 and S5, Supplementary Material online.

We generated a Bayesian phylogeny to place all mammoths into a phylogenetic context. We assigned tip ages based on either median calibrated radiocarbon date or, for samples that were single-sample dated, using the mean point estimate and 95% HPD credibility interval as uncertainty (Fig. 2). The two previously published EP mammoths (Adycha, Krestovka) were notable for each forming their own highly diverged mitochondrial lineages (van der Valk et al. 2021). The two new EP mammoths reported here (P035, P045) both have divergent mitogenome haplotypes that are roughly coeval with Adycha (specimen ages range from 1.20 Ma [95% HPD, 1.50 to 0.90 Ma] to 1.05 Ma [95% HPD, 1.34 to 0.79 Ma]). However, since the posterior probability support values at the relevant nodes are quite low (<0.22), and the divergence times between them are relatively close, the exact relationships between the three mitogenome lineages (P035, P045, Adycha) remain unresolved.

These EP mitogenome lineages existed prior to the emergence of the three LP mammoth mitogenomic clades, which according to our analyses shared a most recent common ancestor (MRCA) around 787 ka (node height 95% HPD, 908 to 792 ka), placing the emergence of LP mammoth mitogenome diversity near the EP-MP boundary (780 ka). Chang et al. (2017) estimated this MRCA range between 2 and 1 Ma, but both their implementation of multi-sample dating and the use of an older node calibration prior to the divergence between Asian elephants and mammoths (at 6.7 Ma) to infer the joint phylogeny, as compared to ours (5.3 Ma; van der Valk et al. (2021)), likely explain their older node age estimate and wider HPD range. It is notable that our estimate for the emergence of the three clades coincides with the paleontological estimate of 800 to 600 ka for the origin of the woolly mammoth as a morphologically distinct species (Lister and Sher 2001).

All mammoths tip-dated to the MP fall within the mitogenomic diversity represented by LP mammoths. In addition to Chukochya falling at the base of Clade 3, we recover two additional MP mammoth mitogenomes (L169 and L171; ∼500 ka) that form a lineage near the base of this Clade—diverging after Chukochya (supplementary fig. S4, Supplementary Material online). Our tip dating estimates also suggest that two previously published mitogenomes, an MP mammoth (SP1145) **from the Altai (Russia)** and a > 50 ka mammoth (SP1785) **from China**, fall respectively, at the base of each of the subclades that comprise European Clade 3 woolly mammoths.

Furthermore, a North American late MP woolly mammoth (MD228; supplementary Text S3, Supplementary Material online) from the Old Crow River in northern Yukon, Canada, which is single-sample dated to 216 ka (95% HPD, 312 to 124 ka), is recovered at the base of all North American Clade 3 mammoths (supplementary fig. S4, Supplementary Material online). This mitogenome represents to our knowledge the oldest DNA so far sequenced from a North American mammoth, suggesting that woolly mammoths reached North America prior to 200 ka. Moreover, it is likely that Clade 3 had a long-term presence in northern Yukon, since all Clade 3 North American mammoths were found in this region, including the only two MP mammoths from North America with available mitogenomes to date (supplementary fig. S4, Supplementary Material online), two mammoths dating to >50 ka according to their mean tip estimates (ranging from 92 to 58 ka; supplementary table S3, Supplementary Material online) and one individual (SEP66) radiocarbon dated to ∼43 ka (Debruyne et al. 2008). Interestingly, the remaining 68 North American mitogenomes included in our analyses date to the LP and belong to Clade 1, including six mitogenomes with tip dates >50 ka.

Our results strongly suggest a Siberian origin of Clade 3. This is supported not only by the fact that the earliest confirmed woolly mammoth fossil in North America dates to ∼220 ka (results above), but also by the earliest proposed arrival of woolly mammoths in Europe based on the paleontological record at around 250 ka (Lister 2022).

Similarly, we identified three MP Siberian mammoths that are basal to Clade 2 (supplementary fig. S4, Supplementary Material online). While the middle MP sample L168 (452 ka; 95% HPD, 604 to 314 ka) is recovered at the base of the Clade, two late MP samples, P048 (247 ka; 95% HPD, 332 to 166 ka) and L172 (196 ka; 95% HPD, 284–111 ka), are basal to all remaining and more recent Clade 2 mammoths. Additionally, all available mammoth mitogenomes assigned to this Clade (*N* = 19; including seven MP mammoths) have a Siberian origin. These results indicate that Clade 2 also originated in Siberia.

A previous study on whole-genome data from two Siberian woolly mammoths, which lived in different time periods (∼45 and ∼4 ka, respectively) and belonged to different mitochondrial clades, identified a marked demographic bottleneck during the MP with a mean estimate of ∼285 ka (Palkopoulou et al. 2015). We note that, within each clade, very few mitogenomes sampled after this date seem to coalesce before ∼285 ka (Figs. 2, supplementary S4 and S5, Supplementary Material online). Furthermore, the diversification of major LP mitochondrial lineages, such as the Eurasian and Siberian branches of Clade 1 (∼280 ka; 95% HPD, 345 to 265 ka) occurred after ∼285 ka (supplementary fig. S5, Supplementary Material online). Considering the geographical and temporal extent—and large sample size—of our mitogenomic dataset, our results are consistent with the proposed demographic bottleneck, due to either declines in population sizes or major population splits, since under both scenarios effective population sizes are expected to decrease. Therefore, we hypothesize that this MP population bottleneck could be linked to major demographic changes given the substantial diversification across all Clades that seems to occur around this period. It is worth noting that most of the subclades also diversify at the MP-LP boundary around the Eemian interglacial, which could be consistent with a bottleneck followed by demographic expansion at the onset of the last glaciation, which has previously been suggested by Palkopoulou et al. (2013).

Finally, we report a new Siberian Clade 1 mitogenome (L088) dated to 116 ka (95% HPD, 151 to 82 ka), near the MP-LP transition (supplementary fig. S5, Supplementary Material online). This individual clusters with three younger mammoths (radiocarbon dated between 24 ka and 38 ka) from eastern Chukotka, the Siberian gateway to the Bering Land Bridge. This cluster falls at the base of the Eurasian branch of Clade 1, potentially supporting an out-of-America hypothesis for this branch, which is based on the hypothetical long-term presence of Clade 1 in North America (Debruyne et al. 2008; Enk et al. 2016; van der Valk et al. 2021). However, current data do not permit us to infer whether this Clade originated in Siberia, Beringia, or North America. Additional deep-time specimens from near the Bering Strait could help answer this long-standing question. Interestingly, all >50 ka Clade 1 LP mammoths from North America (*N* = 6) and Siberia (*N* = 6) are spread across the Clade, falling at the base of main LP lineages (supplementary fig. S5, Supplementary Material online).

Overall, this study showcases how serially sampled mitogenomes across a million-year time scale can provide novel insights into the evolutionary history of a species. The inclusion of deep-time mitogenomes in phylogenetic analyses enables a more precise estimation of basal coalescence times across mitochondrial lineages, which can aid understanding of demographic changes through time. Our reconstruction of mammoth mitogenome phylogeny across the second half of the Pleistocene epoch has provided a glimpse into mammoth diversity during the EP, prior to the diversification of the three Clades characteristic of LP mammoths. Furthermore, having access to MP mitogenomes provided further insights into demographic changes and population dynamics during this stage, as well as the potential impact of such changes on LP mitogenome diversity. For instance, we were able to detect mitogenome diversification events that coincide with major biogeographic changes, such as the EP-MP transition, and a substantial MP demographic bottleneck previously detected using genome-wide data (Palkopoulou et al. 2015). Finally, we provide further insights into long-standing questions such as the appearance of mammoths in North America and the likely North American origin of Clade 1.

On a broader scale, these findings are of interest to the fields of ancient DNA as well as phylogeography and paleontology. First, our results highlight the feasibility of using the molecular clock to estimate the age of samples that are far beyond the limits of radiocarbon dating and improve previous approaches to perform these estimations. Importantly, we confirm that there is a bias when simultaneously estimating the age of multiple samples at the same time using tip-calibrated dating in BEAST (supplementary fig. S2, Supplementary Material online), as previously suggested (Karpinski et al. 2020). We therefore recommend that tip-calibrated dating is performed on one undated sample at a time. Second, we demonstrate that novel biogeographic insights can be gained from generating temporal mitogenome transects that extend into the Middle and Early Pleistocene. Temporal mitogenome transects could be used to test key hypotheses in phylogeography, for example, whether species-specific postglacial recolonization routes (Hewitt 2001) are congruent across multiple glacial cycles, and to what extent refugial populations display genetic continuity through time. Finally, we note that the possibility of recovering MP genome-wide data will provide a unique opportunity to investigate whether, and if so which, species went through demographic bottlenecks during previous interglacial periods, considering that this data will provide a wider representation of the genetic diversity of the species at different points in time. This could have far-reaching implications for our understanding of the impact of climate change on species evolution.

## Materials and Methods

### Sample Collection and Mitogenomic Data Generation

The samples used to generate the 34 new mitogenomes reported in this study have been collected over several decades from different museum collections and field trips. These samples were processed in different laboratories; seven samples at the Centre for Palaeogenetics (Stockholm, Sweden) and 27 samples at the Ancient Biomolecules Lab, University of York (York, UK) and the Adaptive Genomics Lab, University of Potsdam, (Potsdam, Germany) as described below (see supplementary table S1, Supplementary Material online for details).

Stockholm: We first collected 50 to 200 mg of bone or tooth powder per sample using a Dremel drill. DNA was extracted for each sample following the protocol in Dabney et al. (2013). For two of these samples (MD228 and MD229) extractions were slightly modified to follow the final silica column protocol presented in Dehasque et al. (2022). We prepared double-stranded sequencing libraries following Meyer and Kircher (2010), including treatment with either 3 or 6 uL of USER enzyme (New England Biolabs) to excise uracil bases incorporated due to post-mortem DNA damage as described by Dehasque et al. (2022). All clean-up steps were performed using MinElute purification columns (QIAGEN). PCR reaction conditions were the same as described by Pečnerová et al. (2017). The number of PCR cycles varied from 6 to 16, depending on DNA library quantity as indicated by the Ct values of a qPCR. We generated several independent amplified libraries for each sample to minimize sequence clonality during sequencing. We removed both too-short (bead-to-mix ratio, 1.8) and too-long fragments (bead-to-mix ratio, 0.5) from the amplified libraries using magnetic Agencourt AMPure XP beads (Beckman Coulter). The final amplified libraries were sent to the National Genomics Infrastructure (NGI Stockholm) for shotgun sequencing using the Illumina NovaSeq platform (supplementary S4, Supplementary Material online lane with either 2 × 100 or 2 × 150 bp). Negative controls were included at each step (DNA extraction, library preparation, and PCR).

York/Potsdam: For each sample, a large area of tusk or bone was first cleaned with a Dremel drill bit, then a small piece was removed and ground with a pestle and mortar to generate approximately 50 mg of powder per sample. DNA was extracted for each sample following the protocol by Dabney et al. (2013). We prepared single-stranded sequencing libraries following the protocol of Gansauge and Meyer (2013) with additional modifications (Korlević et al. 2015; Basler et al. 2017) including the use of afu uracil-DNA glycosylase (UDG) to remove uracils at non-terminal base positions resulting from cytosine deamination. The optimal number of PCR cycles was estimated prior to indexing amplification using qPCR as above. Post-amplification purification was performed using Qiagen MinElute columns. Each library was quantified prior to sequencing by measuring the DNA fragment size on a Tapestation 2200 (Agilent Technologies) using the D1000 ScreenTape System, and the library concentration was assessed on a Qubit Fluorometer (ThermoFisher Scientific). Libraries were enriched for mitochondrial DNA by performing two rounds of in-solution hybridization capture, described by González Fortes and Paijmans (2019) using the elephantid mitochondrial capture baits from Meyer et al. (2017). Samples were pooled prior to paired-end sequencing (2 × 50 bp), which was performed on one lane of an Illumina HiSeq-2000/2500 v4 high-output platform at the Genepool Sequencing Facility at the University of Edinburgh, using a custom sequencing primer (Paijmans et al. 2017). Negative controls were included at each step (DNA extraction, library preparation, and PCR).

## Data Analysis

### Raw Data Processing, Iterative Assembly, and Consensus Calling

Raw FASTQ sequencing data files for the 34 samples were adapter trimmed and overlapping paired-end reads were merged using fastp v0.11.0 (Chen et al. 2018) with default settings, only keeping merged reads longer than 35 bp to reduce misalignments during the iterative assembly step (van der Valk et al. 2021). Mitogenomes were assembled using the mapping-iterative assembler (MIA) (Green et al. 2008) with the Asian elephant (*Elephas maximus*) reference mitogenome (NC_005129) as a guide. Positions with less than 3× coverage or with a sequence agreement of less than 67% were marked as missing data using custom python scripts (https://github.com/aersoares81/mia-helper-scripts). Depth and breadth of coverage data for all consensus sequences can be found in supplementary table S1, Supplementary Material online. Khroma, a sample previously shotgun sequenced but without a mitogenome assembly (Seguin-Orlando et al. 2015) was assembled in the same way but the quality filtering for calling a consensus sequence was more strict to account for deamination damage. In this case, positions with less than 10× coverage or with a sequence agreement of less than 90% were marked as missing data. Depth and breadth of coverage for this mitogenome are 312.6× and 99.8%, respectively.

### Multiple Sequence Alignment of New and Previously Published Mitogenomes

The original alignment published by van der Valk et al. (2021) was unaligned by removing sequence gaps with a custom python script (https://github.com/jcchacond/unalignMSA). Four straight-tusked elephants (*Palaeoloxodon antiquus*) included in this dataset were removed since they were not used in any downstream analyses in the present study, in order to avoid having undated samples in the reference dataset for tip calibration. This multi-sample FASTA file was then merged with the newly generated mitogenomes (including the one generated from the sequencing data from the study by Seguin-Orlando et al. (2015). Additionally, we added 20 mammoth mitogenomes from Fellows-Yates et al. (2017) to increase the geographical representation of Europe. Finally, a multiple sequence alignment (MSA) was performed with MUSCLE v3.8.31 (Edgar 2004) using default parameters.

SeaView v5.0.5 (Gouy et al. 2021) was used to visually inspect the resulting MSA. Bases supported by only one sample (and assigned as gaps in the remaining samples) were removed. Liftoff v1.6.3 (Shumate and Salzberg 2021) was used to translate the woolly mammoth reference mitogenome annotation to the new coordinates of this mitogenome in the MSA. Considering the assembly and alignment limitations on the hypervariable control region, the lower and upper limits of the variable number tandem repeat (VNTR) region (positions 16157 to 16476 in the woolly reference mitogenome; NC_007596) were located in the MSA and added to the output annotation file in order to mask it for downstream analyses.

### Single-sample Molecular Clock Dating

As described in the Results section, we performed molecular clock tip dating using the Bayesian approach first described by Shapiro et al. (2011), which is implemented in the software BEAST v1.10.4 (Drummond and Rambaut 2007), following the methodology implemented by van der Valk et al. (2021). First, we replicated this methodology using their own MSA (downloaded from https://palaeogenetics.com/data-and-scripts/). Then, after running a series of tests we modified this methodology to perform single-sample tip dating (see Results and supplementary Text S1, Supplementary Material online). We generated a molecular dating reference dataset for tip calibration that only contains mammoth samples with finite radiocarbon ages and modern-day mitogenomes from both Asian (*Elephas maximus*) and African elephants (*Loxodonta africana, Loxodonta cyclotis*) as outgroups, for a total of 153 mitogenomes. All radiocarbon dates were recalibrated to the IntCal20 terrestrial calibration curve (Reimer et al. 2020) using OxCal (Ramsey 1995) (supplementary tables S1 and S2, Supplementary Material online), and the median recalibrated value was used for all downstream analyses.

Each of the samples without a finite radiocarbon age was merged, one at a time, with the reference dataset, for a total of 75 single-sample dating datasets. The same procedure was followed for the eight radiocarbon dated samples used to test the single-sample dating approach (supplementary Text S2, Supplementary Material online). Following van der Valk et al. (2021), the alignment was split into six partitions: tRNA, rRNA, first, second, and third codon positions, and the control region. All partitions were assigned the HKY + Gamma + Invariant substitution model, except for the tRNA partition, for which the HKY + Invariant model was used. We used the same log-normal prior for the divergence between *Loxodonta* and *Elephas/Mammuthus* of 5.3 Ma with a strict molecular clock and a flexible skygrid coalescent model (van der Valk et al. 2021). For the undated mitogenomes, we used a uniform tip prior with a range of 1 ka to 2 Ma (see Results and supplementary Text S1, Supplementary Material online for details). In order to generate the input files for each single-sample dating dataset we used a custom Python script (https://github.com/VanssyLi/XMLgenerator_v1).

For each dataset, we ran two independent MCMC chains in BEAST for 100 million iterations each, sampling every 10,000 and discarding the first 10% as burn-in. We checked for convergence between the run pairs as well as an uni-modal posterior distribution of the age estimates using Tracer v1.7.2 (Rambaut et al. 2018). A custom Python script (https://github.com/VanssyLi/BEASTLogParsing) was used to parse the output and extract the tip date estimations, their 95% HPD intervals, and the effective sample size (ESS) values to ensure an adequate amount of MCMC sampling (ESS >200). Every pair of mean estimates and their respective 95% high posterior density values were averaged to obtain a unique age estimate per sample (supplementary table S3, Supplementary Material online).

### Joint Phylogeny With Estimated Molecular Tip-dates as Input

We merged all single-sample dated mitogenomes with the molecular dating reference dataset MSA for a total of 236 mitogenomes (225 mammoths and 11 elephants). For the single-sample dated mitogenomes, we used the mean molecular tip-dated age as the age of the sample and the range of dates covered by the 95% HPD as uncertainty. We ran two independent MCMC chains of 100 million iterations in BEAST, with the same parameters as described earlier. The final Maximum Clade Credibility tree was extracted from the BEAST output and visually inspected with the BEAST tools TreeAnnotator v1.10 and FigTree v1.4.4, respectively. The final trees for the paper figures were produced using R v4.3.2 (R Core Team 2021), RStudio v2024.04.1 (RStudio Team 2020), and the *Tidyverse* v2.0.0 set of packages (Wickham et al. 2019), as well as the packages *treeio* v1.26.0 (Wang et al. 2020), *ggtree* v3.10.0 (Yu et al. 2017), and *ape* v5.7-1 (Paradis and Schliep 2019).

## Supplementary Material

msaf065_Supplementary_Data

## Data Availability

All newly assembled mitogenomes, together with those from van der Valk et al. (2021), are deposited in GenBank (accession numbers PV138979 to PV139017).
