# Supplementary material for: A Million Years of Mammoth Mitogenome Evolution
Source: Mol Biol Evol. 2025 Apr 9;42(4):msaf065. doi: 10.1093/molbev/msaf065 (PMC11980863; doi:10.1093/molbev/msaf065)
Supplement: msaf065_Supplementary_Data [file msaf065_supplementary_data.zip › Chacon-Duque-etal-2025_MBE_Suppl-mat_final.pdf]

Supplementary material

A million years of mammoth mitogenome evolution

JC Chacón-Duque, JA Thomas Thorpe, *et al*.

List of Supplementary Figures

Figure S1. Comparison of multi-sample dating and single-sample dating approaches for the three deep-time samples published in van der Valk et al. (2021)

Figure S2. Tip-dating biases when sequentially adding the five oldest samples for multi-sample dating

Figure S3. Age estimates of 24 Late Pleistocene mitogenomes (<126 ka) that are beyond the limit of radiocarbon dating (>50 ka)

Figure S4. Joint phylogenetic tree zooming in into Clades 2 and 3

Figure S5. Joint phylogenetic tree zooming in into Clade 1

List of Supplementary tables (attached separately as an excel file)

Table S1. Newly sequenced sample metadata

Table S2. Metadata for previously published mitogenomes included in this study

Table S3. Inferred ages for the undated samples using the single-sample dating approach

List of Supplementary Texts

Text S1. Additional information about the implemented single-sample dating approach

Text S2. Testing the single-sample dating approach with radiocarbon dated samples

Text S3. Old Crow mammoth (MD228)

Figure S1. Comparison of multi-sample dating and single-sample dating approaches for the three deep-time samples published in van der Valk et al. (2021)

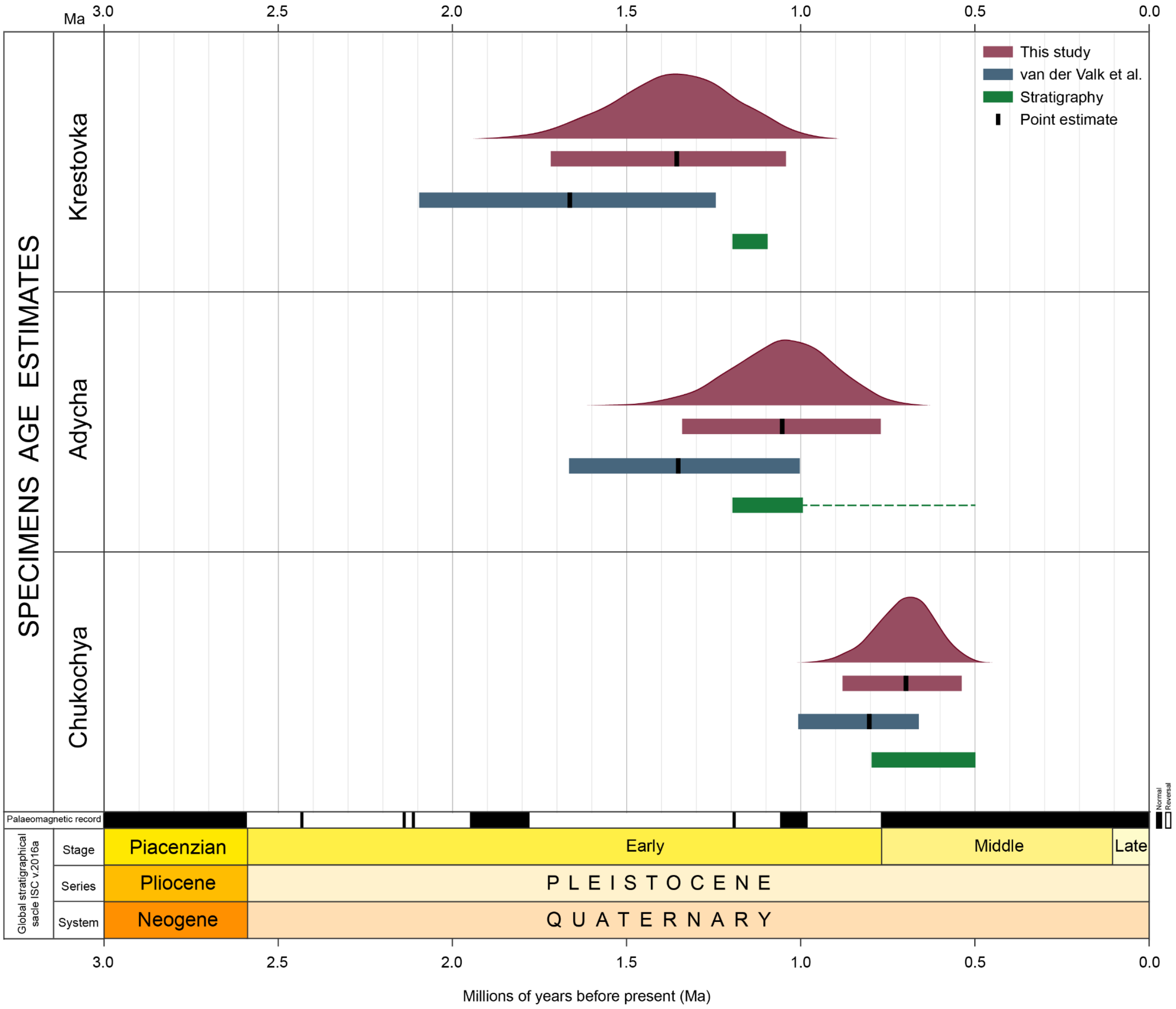

"This study" refers to the single-sample dating approach and "van der Valk et al." to their multi-sample dating approach (van der Valk et al. 2021). A table with these results can be found in Text S1.

Figure S2. Tip-dating biases when sequentially adding the five oldest samples for multi-sample dating

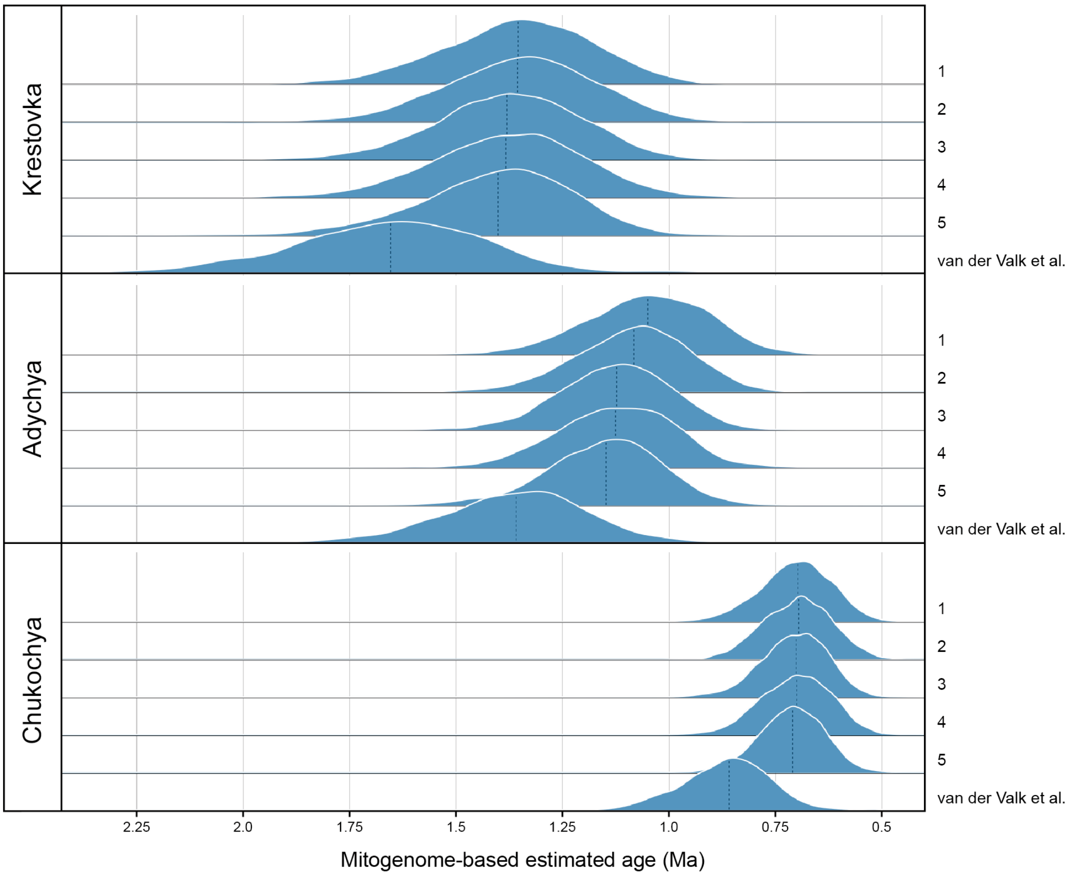

Numbers (1-5) refer to the number of undated specimens included in each independent analysis. Details about the samples used can be found in Text S1. The analysis performed in van der Valk et al. (2021) simultaneously tip-dated 54 specimens, which likely explains the difference of this result with the other five.

Figure S3. Age estimates of 24 Late Pleistocene mitogenomes (<126 ka) that are beyond the limit of radiocarbon dating (>50 ka)

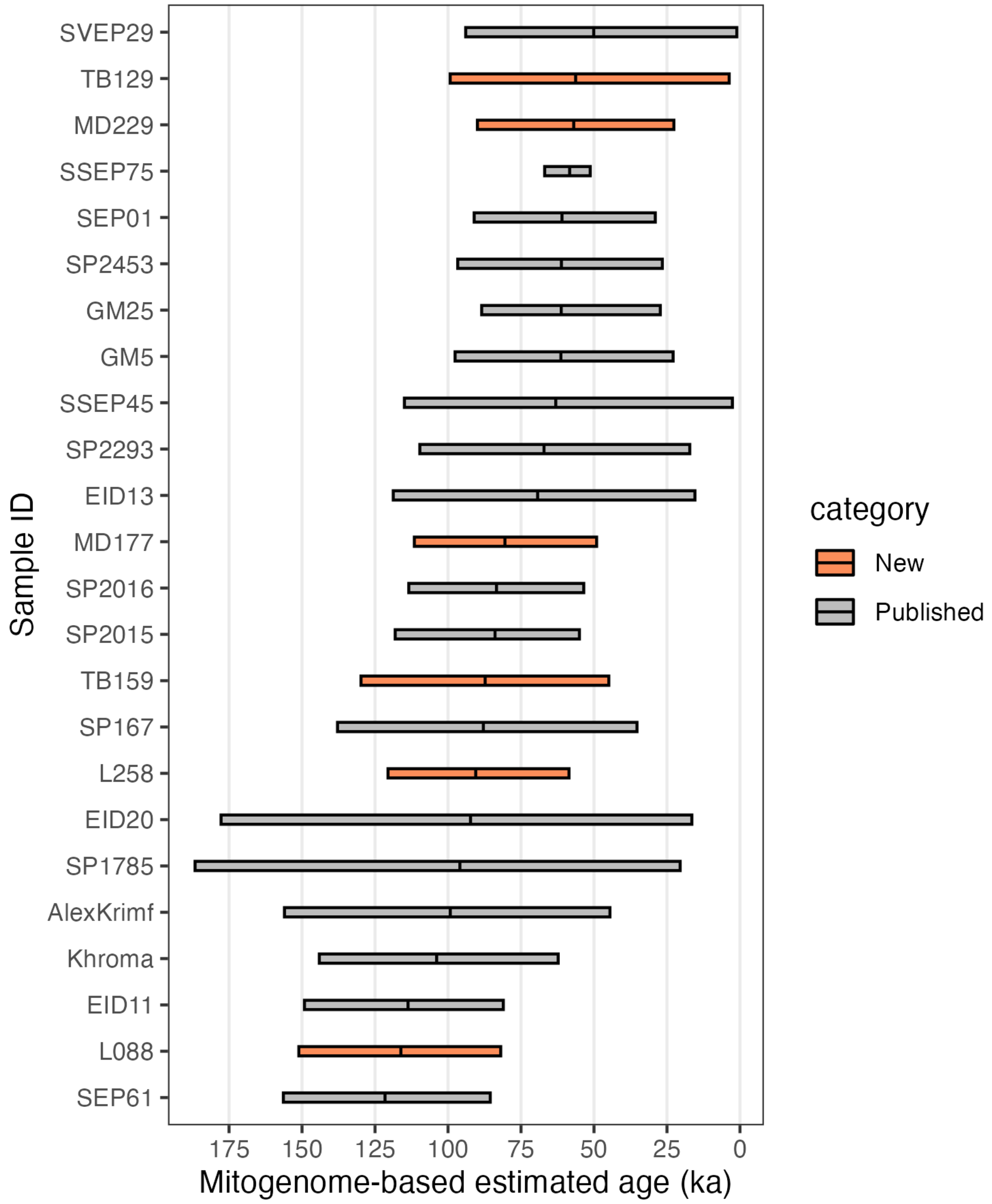

Each bar displays the 95% Highest Posterior Density (HPD) interval and the black line shows the mean estimate.

Figure S4. Joint phylogeny zooming in into Clades 2 and 3

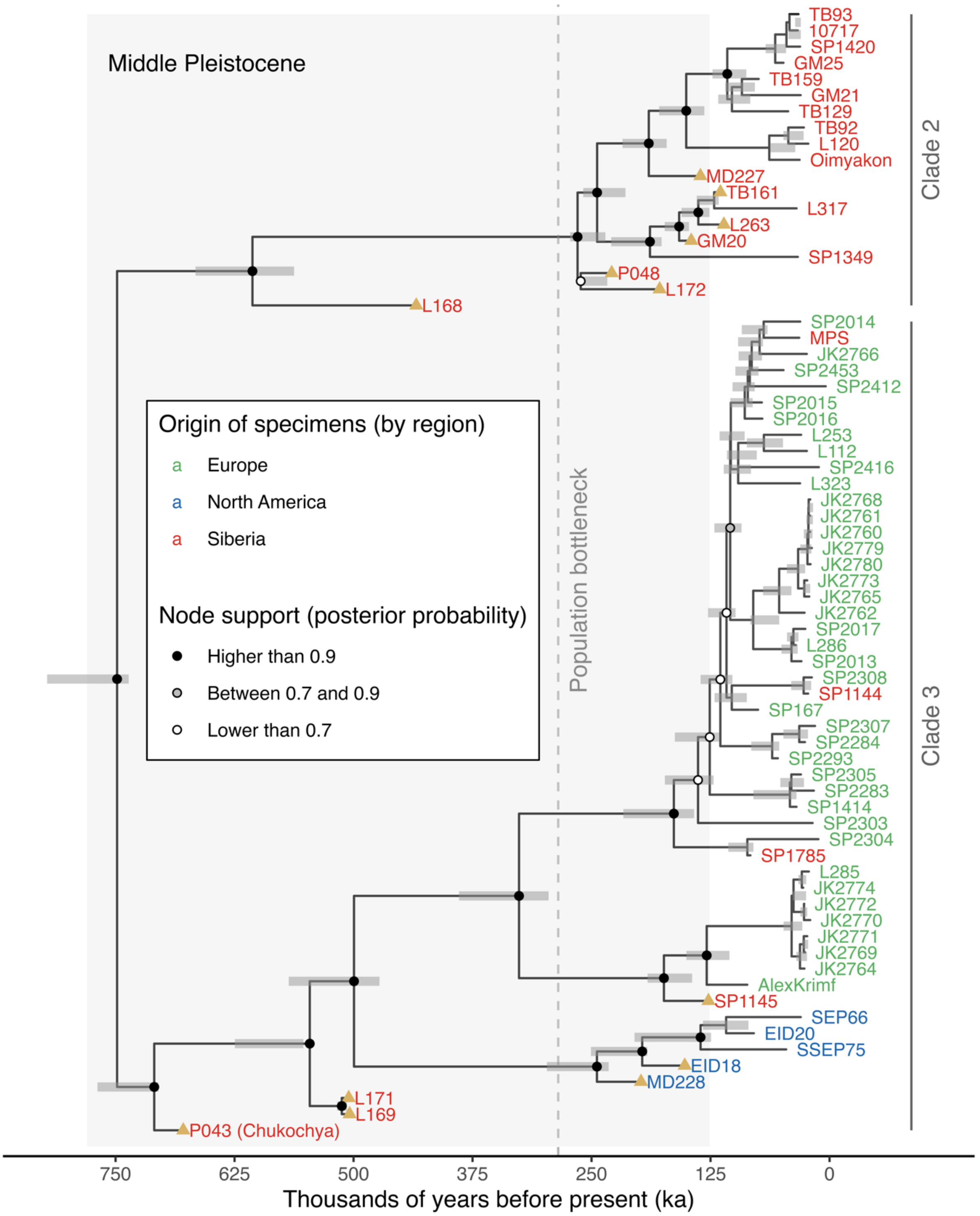

Joint phylogeny using single-sample dating estimates as input for tip ages. The grey shaded area represents the Middle Pleistocene epoch and the dashed line the mean estimate for the demographic bottleneck inferred with genome-wide data (Palkopoulou et al. 2015). The dark grey shades in the nodes display the 95% HPD node heights estimated by the Bayesian analyses. Deep-time specimens are highlighted with a golden triangle.

Figure S5. Joint phylogeny zooming in into Clade 1

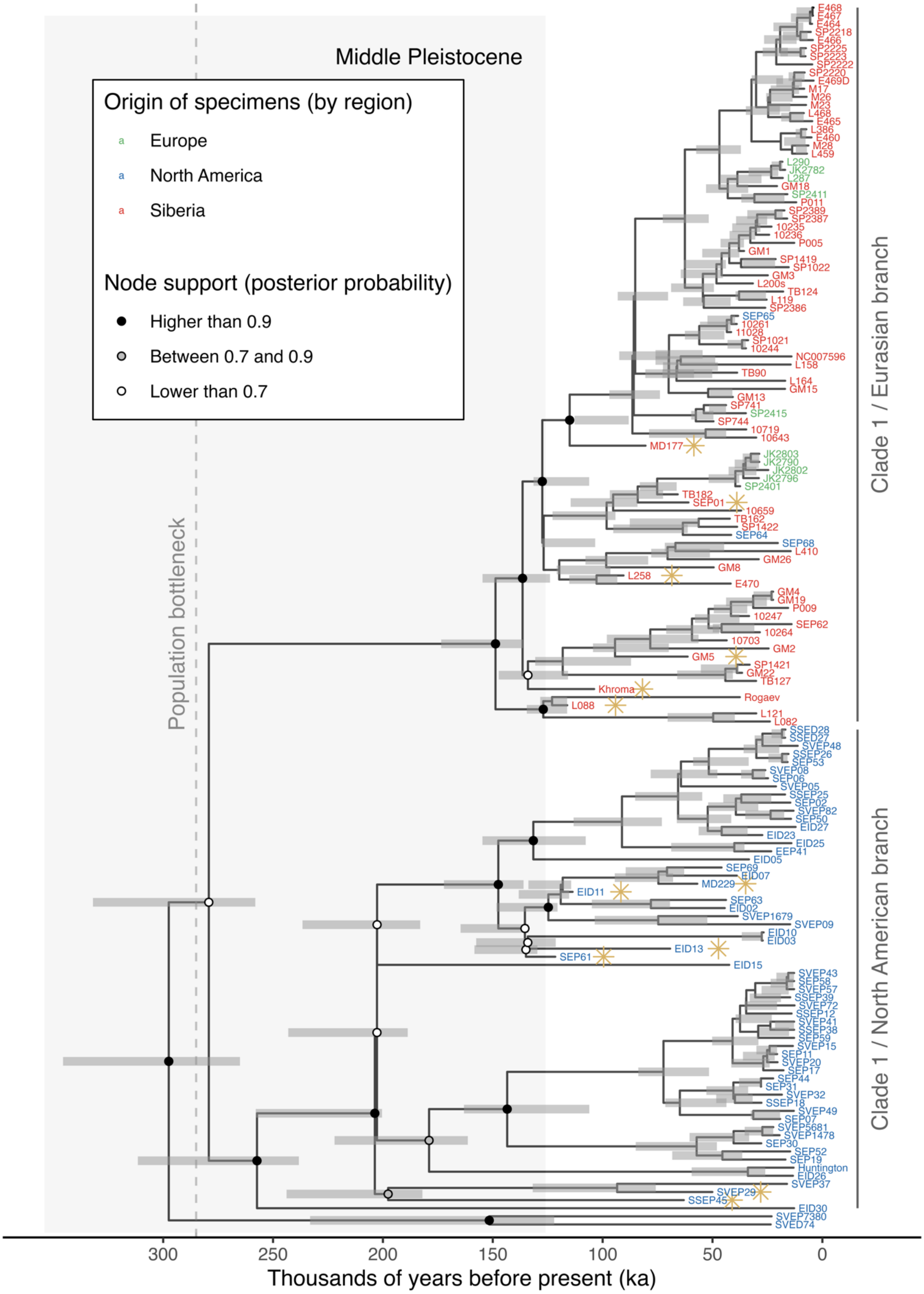

Joint phylogeny using single-sample dating estimates as input for tip ages. The oldest Late Pleistocene specimen new to this paper (L088) as well as the other LP Clade 1 mammoths >50 ka are highlighted with a golden asterisk. See more details in Figure S4.

Text S1. Additional information about the implemented single-sample dating approach

Closer agreement between single-sample tip dated specimens and their stratigraphic context

As shown in Figure S1, we found a better agreement between the single-sample dating estimates and the stratigraphic information, compared to those obtained by van der Valk et al. (2021):

Age estimates of the deep-time mitogenomes presented in Figure S1

Specimen ID	Mean age estimate (95% HPD range)		
	This study	van der Valk et al.	Stratigraphy
Krestovka	1.36 Ma (1.02 - 1.71)	1.65 Ma (1.25 - 2.08)	1.1 - 1.2 Ma
Adycha	1.05 Ma (0.79 - 1.35)	1.34 Ma (1.06 - 1.69)	1.0 - 1.2 Ma
Chukochya	0.70 Ma (0.54 - 0.87)	0.87 Ma (0.68 - 1.07)	0.5 -0.8 Ma

Moreover, nine out of the 11 new mitogenomes that are tip dated to the Early (EP) and Middle Pleistocene (MP) (Table S3) were obtained from specimens dating back to these stages according to their stratigraphy contexts (Table S1). The degree of agreement between molecular and stratigraphic age estimates likely depends on the amount of stratigraphic information available for each specimen. Some cases to highlight include:

- P035, an EP *M. trogontherii*-like mammoth hypothesised to be of Early Olyorian age (~1.2 – 0.8 Ma), which yielded a mean tip-dating estimate of 1.20 Ma.

- MD228, the oldest North American mammoth mitogenome reported to date, which was found *in situ* below MP sediments from the Old Crow Tephra (>160 ka) and yielded a molecular tip-date mean estimate of 216 ka (see Text S3 for a full description of this specimen).

Effect of sequentially adding undated samples to the multi-sample tip dating approach

Additionally, we demonstrated the effect of sequentially adding the five oldest undated mitogenomes to the analyses: Krestovka, Adycha, Chukochya, EID18 and GM20. For instance, in the case of Chukochya we used the following combinations: 1-sample dating: Chukochya; 2-sample dating: Chukochya and Krestovka; 3-sample dating: Chukochya, Krestovka, and Adycha; 4-sample dating: Chukochya, Krestovka, Adycha and EID18; 5-sample dating: Chukochya, Krestovka, Adycha and EID18 and GM20.

The following table contains the results presented in Figure S2:

Age estimates of multiple-sample dating analyses sequentially adding the five oldest undated samples

Multiple-sample analyses	Mean age estimate (95% HPD range)		
	Krestovka	Adycha	Chukochya
1-sample dating	1.36 Ma (1.02 - 1.71)	1.06 Ma (0.79 - 1.34)	0.70 Ma (0.54 - 0.87)
2-sample dating	1.36 Ma (1.01 - 1.69)	1.09 Ma (0.84 - 1.36)	0.70 Ma (0.54 - 0.86)
3-sample dating	1.38 Ma (1.06 - 1.73)	1.13 Ma (0.88 - 1.38)	0.70 Ma (0.56 - 0.86)
4-sample dating	1.38 Ma (1.04 -1.74)	1.13 Ma (0.87 - 1.40)	0.70 Ma (0.55 - 0.86)
5-sample dating	1.41 Ma (1.09 - 1.78)	1.15 Ma (0.88 - 1.41)	0.71 Ma (0.58 - 0.88)
van der Valk et al.	1.65 Ma (1.25 - 2.08)	1.34 Ma (1.06 - 1.69)	0.87 Ma (0.68 - 1.07)

Assessment of different age prior distributions

Considering that for deep-time samples stratigraphy-based age estimates are often very broad and that in some cases it is not possible to guess the age of a specimen (e.g. specimens found ex situ), the selection of appropriate tip priors is important. Using the single-sample dating approach and the same parameters described in the main text, we tip-dated three sets of samples using three types of prior distributions (i) log-normal as used by van der Valk et al. (ii) normal and (iii) uniform.

The results below show that uniform and log-normal distribution priors produce similar posterior probabilities, while the normal distribution prior is constrained by the input, hence tending to get narrower estimates that are usually biassed towards the parameter provided. These results suggest that normal priors should be used with caution, and it could be a good practice to compare results obtained with several age prior distributions.

Age estimates using three different tip priors

**Specimen ID**	**Mean age estimate (95% HPD range)**		
	**log-normal**	**normal**	**uniform**
Krestovka	1.36 Ma (1.02 - 1.71)	1.10 Ma (0.94 - 1.26)	1.42 Ma (1.06 - 1.81)
Adycha	1.05 Ma (0.79 - 1.35)	1.02 Ma (0.86 - 1.17)	1.08 Ma (0.79 - 1.40)
Chukochya	0.70 Ma (0.54 - 0.87)	0.84 Ma (0.71 - 1.00)	0.70 Ma (0.55 - 0.87)
KX027565	121.52 ka (83.28 - 160.28)	105.55 ka (87.78 - 122.65)	125.67 ka (89.92 - 167.53)
KX176770	90.73 ka (45.14 - 136.12)	100.06 ka (82.67 - 117.90)	98.72 ka (51.40 - 145.67)
KX176774	79.05 ka (45.78 - 112.78)	94.88 ka (78.60 - 112.18)	80.39 ka (43.98 - 114.08)
EU153452	40.22 ka (16.00 - 67.49)	45.27 ka (29.30 -61.58)	38.48 ka (10.45 - 62.52)
KX176788	23.61 ka (7.99 - 39.30)	33.83 ka (19.76 - 47.95)	21.76 ka (10.01 - 35.34)
KX176791	26.24 ka (6.62 - 51.44)	42.13 ka (24.82 - 60.14)	24.10 ka (10.00 - 47.34)

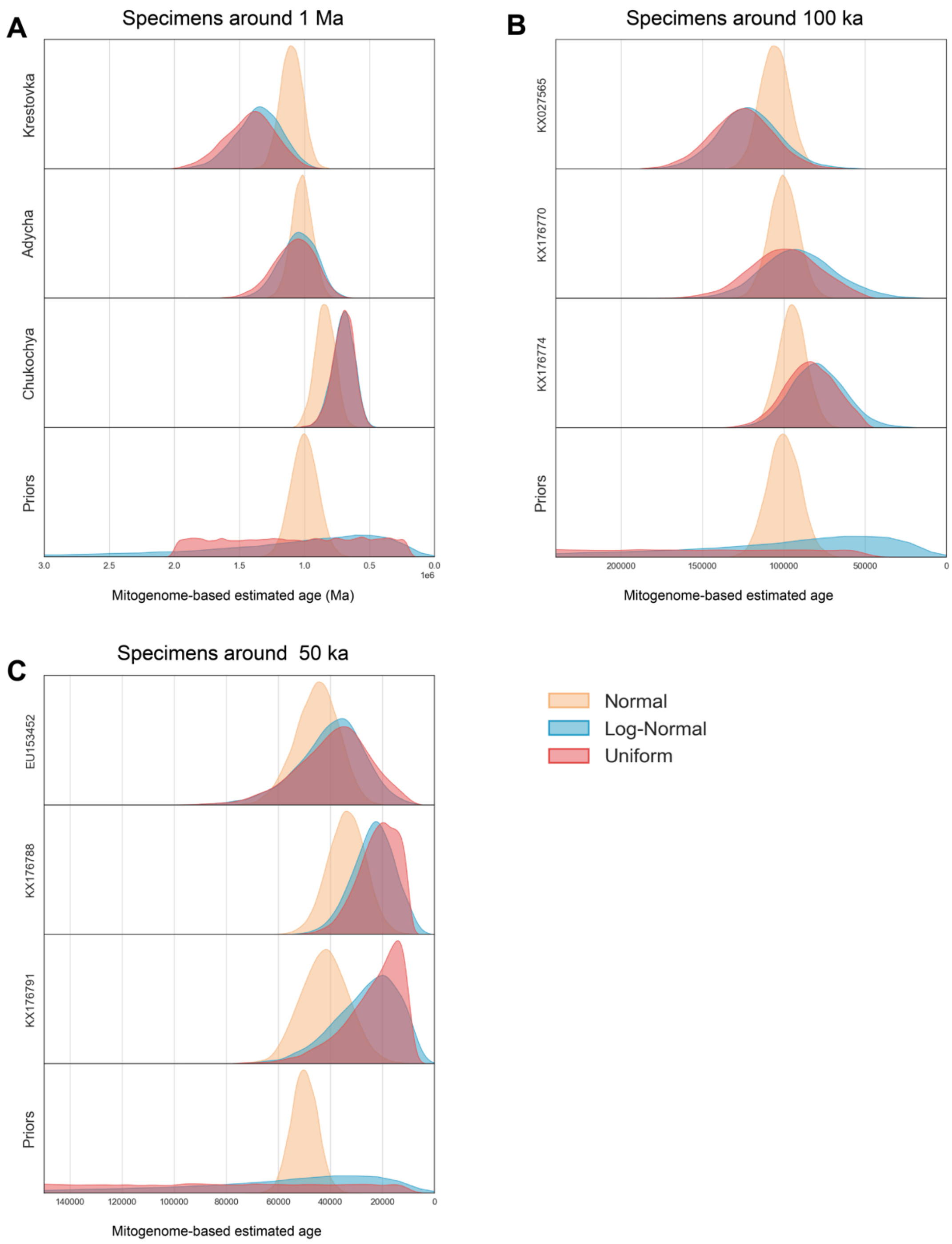

Mitogenome-based age estimation of specimens >50 ka. **A)** Specimens ~1 Ma with log-normal (mu=13.8, sigma=0.8, offset=0.0), normal (mean=1,000,000, stdev=100,000) and uniform priors (lower=200,000, upper=2,000,000). **B)** Specimens around ~100 ka with log-normal (mu=11.5, sigma=0.87, offset=0.0), normal (mean=100,000, stdev=10,000) and uniform priors (lower=50,000, upper=500,000). **C)** Specimens ~50 ka with log-normal (mu=11.5, sigma=0.87, offset=0.0), normal (mean=50,000, stdev=5,000) and uniform priors (lower=10,000, upper=200,000). The last panel on each figure represent the prior distributions that were provided for the Bayesian analysis.

Text S2. Testing the single-sample dating approach with radiocarbon dated samples

To test the accuracy and precision of single-sample molecular tip dating, especially in more recent samples, we estimated the ages of eight samples with finite radiocarbon estimates which were not included as tip calibration reference sequences.

Single-sample tip dating estimates for samples with known radiocarbon ages

Lab ID	Clade	Region	Age estimate	
			Median calibrated age IntCal 20	Tip-dating Mean estimate (95% HPD range)
L119	1	Siberia	25.40 ka	18.89 (1.01 - 41.21) ka
L121	1	Siberia	30.12 ka	29.66 (1.03 - 60.12) ka
L323	3	Europe	49.14 ka	43.77 (1.13 - 77.30) ka
TB124	1	Siberia	17.87 ka	16.09 (1.00 - 36.96) ka
TB127	1	Siberia	30.19 ka	33.83 (1.04 - 62.76) ka
TB162	1	Siberia	42.17 ka	66.14 (32.28 - 99.54) ka
TB182	1	Siberia	49.39 ka	65.84 (38.92 - 92.92) ka
TB90	1	Siberia	38.81 ka	62.88 (41.37 - 85.33) ka

Results for the first five samples in the table show that mean tip-dating estimates are generally congruent with median calibrated radiocarbon estimates, despite the wide 95% High Posterior Density (HPD) ranges. This suggests that even though molecular tip dating cannot be used as a replacement for radiocarbon dating, it could potentially be used for exploratory analyses, at least when there is evidence that the tip calibration reference sequences provide a strong temporal signal.

It is also important to note that the samples TB162, TB182 and TB90 don’t show the same congruence between different estimates, but for two out of three cases, the radiocarbon date is covered within the lower bound of the 95% HPD range in the tip dating. This inconsistency could be related to these samples being close to the limit of radiocarbon dating and/or being contaminated by younger material during the radiocarbon dating process, but further testing will be needed to draw any conclusions on this regard.

Text S3. Old Crow mammoth (MD228)

The mitogenome corresponding to sample MD228 was obtained from a left molar rest (second upper M2 (M2. sup. sin.)) from the Yukon Government Palaeontology Program, Whitehorse, Yukon, Canada (Accession number YG 812.20). It was excavated in situ from Pleistocene sediments that are stratigraphically below the Old Crow Tephra (>160 ka) (Reyes et al. 2023) at Old Crow River locality CR 11 by Grant Zazula and Greer Vanderbyl (July 24, 2018). Previous work at the locality has documented in situ fossils of various mammals, including mammoths (*Mammuthus* sp.), horse (*Equus*), caribou (*Rangifer tarandus*), giant beaver (*Castoroides ohioensis*), and Jefferson's ground sloth (*Megalonyx jeffersonii*), that are correlative to the Marine Isotope Stage 7 interglaciation or older (Froese et al. 2017). Importantly, fossils of steppe bison (*Bison priscus*) are absent from these sediments, suggesting this represents a late Irvingtonian fauna, rather than a late Pleistocene Rancholabrean fauna which are more typical from eastern Beringia. The specimen was classified as a woolly mammoth (*Mammuthus primigenius*; Blumenbach, 1799) as described below:

The overall morphology and number of remaining lamellae indicates it is a penultimate molar, M2. A total of 14 plates are still present and an unknown number of plates are worn away on the mesial side. It displays conspicuous thin lamellae and enamel. The original number of lamellae is no longer present and has been worn away through use on the mesial side, two root bases also missing an unknown number of lamellae, presumably one or two.

Occlusal surface is convex, characteristic of maxillary molars. Buccal side convex, deposited on buccal side a layer of cementum of 5 mm thickness. Lingual side is concave, covered with a thin layer of up to 1 mm thickness of cementum.

The root section of the molar rest is completely resorbed away. What remains of the root parts are remnants recognisable as roots with which the molar was anchored in the maxilla. The surface structure of these root remains is pockmarked. This indicates that the molar was no longer anchored in the jaw. Possibly already fallen out.

Measurements:

Maximum length, measured across the axis of the chewing surface: 123 mm

Maximum width, measured at the centre of the molar, over the chewing surface: 90 mm

Maximum crown height (mesial): 2 mm

Maximum crown height (distal): 42 mm

Maximum enamel thickness: 1.3 mm

Information on individual age: Associated with this is a mandible that has the m2, fully in use and partially used and a m3 partially, ca 50 % in use, Laws Age Class (1966), XIX which indicates an individual age at time of death of approximately 32 African Equivalent Years.

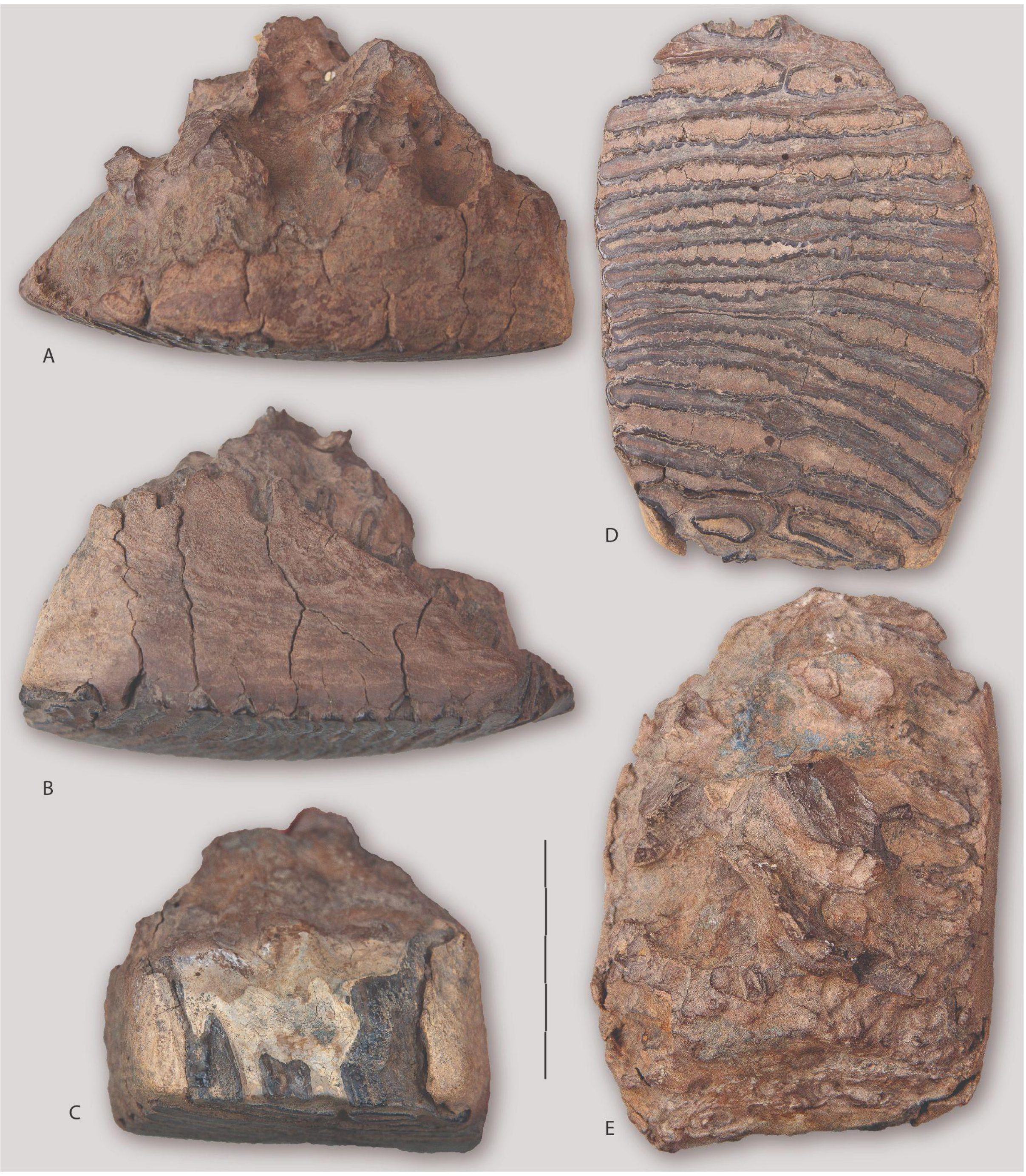

Second upper left molar rest M2 (M2 sup. sin.) *Mammuthus primigenius* (Blumenbach, 1799), Yukon Government Palaeontology Program, accession number YG 812.20. Scale bar: 5 cm. A) lingual view. B) buccal view. C) posterior view. D) occlusal view. E) view from the root section affected by acids from bacteria.

References

Froese D, Stiller M, Heintzman PD, Reyes AV, Zazula GD, Soares AER, Meyer M, Hall E, Jensen BJL, Arnold LJ, et al. 2017. Fossil and genomic evidence constrains the timing of bison arrival in North America. *Proc. Natl. Acad. Sci. U. S. A.* 114:3457–3462.

Palkopoulou E, Mallick S, Skoglund P, Enk J, Rohland N, Li H, Omrak A, Vartanyan S, Poinar H, Götherström A, et al. 2015. Complete genomes reveal signatures of demographic and genetic declines in the woolly mammoth. *Curr. Biol.* 25:1395–1400.

Reyes AV, Jensen BJL, Woudstra SH, Bolton MSM, Buryak SD, Cook MS, Harvey J, Westgate JA. 2023. Detrital glass in a Bering Sea sediment core yields a ca. 160 ka Marine Isotope Stage 6 age for Old Crow tephra. *Geology* 51:106–110.

van der Valk T, Pečnerová P, Díez-del-Molino D, Bergström A, Oppenheimer J, Hartmann S, Xenikoudakis G, Thomas JA, Dehasque M, Sağlıcan E, et al. 2021. Million-year-old DNA sheds light on the genomic history of mammoths. *Nature* 591:265–269.
